# Investigating information needs and preferences regarding digital mental health services among medical and psychology students in Germany: A qualitative study

**DOI:** 10.1177/20552076231173568

**Published:** 2023-05-25

**Authors:** Pia Braun, Ann-Kathrin Schwientek, Peter Angerer, Lisa Guthardt, Andrea Icks, Adrian Loerbroks, Jennifer Apolinário-Hagen

**Affiliations:** 1 Institute of Occupational, Social and Environmental Medicine, Centre for Health and Society, Medical Faculty, 9170 Heinrich Heine University Düsseldorf, Dusseldorf, Germany; 2 Department of Psychiatry and Psychotherapy, University Hospital rechts der Isar, School of Medicine, Technical University of Munich, Munchen, Germany; 3 Institute for Health Services Research and Health Economics, Centre for Health and Society, Medical Faculty, 9170 Heinrich Heine University Düsseldorf, Dusseldorf, Germany

**Keywords:** Digital health, digital health literacy, technology acceptance, mental health, medical students, psychology students

## Abstract

**Background:**

Since 2020, physicians and psychotherapists in Germany can prescribe digital mental health services (dMHSs). However, even future healthcare professionals (HCPs), such as medical and psychology students, remain reluctant to use dMHSs, although they are a risk group for mental health issues themselves. Reasons include scepticism and lacking awareness of dMHSs, which can be addressed by acceptance-facilitating interventions (AFIs) such as information strategies. To date, though, little is known about their information needs.

**Methods:**

Semi-structured interviews with *n* = 21 students were conducted between August and September 2021. Students of legal age studying psychology or medicine at a German university could participate. Interview recordings were transcribed verbatim and content-analyzed according to Mayring, using deductive and inductive coding.

**Results:**

Most students reported having little experience with dMHSs. Digital health has barely been raised in their study, even though it was perceived as crucial for personal needs as well as in preparation for their work as HCPs. Students favoured receiving information on and recommendations for dMHSs from their university via, e.g. social media or seminars. Among others, information about data safety, scientific evidence base and application scope were preferred. Additionally, information on costs as well as user reviews seemed to be essential components of information strategies because students were concerned that high costs or low usability would hinder uptake.

**Conclusions:**

The results give first insights on how future HCPs would like to be informed on dMHSs. Future research should focus on systematic variations of AFIs' components mimicking real-world decision scenarios to increase the adoption of dMHSs.

## Introduction

Common mental disorders (CMDs), such as depression, post-traumatic disorders or anxiety disorders, remain among the top 10 leading causes of burden worldwide, while the prevalence of CMDs seems consistent.^1–3^ However, in the wake of the COVID-19-pandemic, CMDs have increased tremendously.^4,5^ Innovation in digital health tools, including mental health apps, provide new approaches to the management of CMDs. For instance, evidence-based digital mental health services (dMHSs) have been suggested as promising options for the large-scale dissemination of interventions for the prevention and treatment of CMDs.^6,7^ Concerning prevention purposes, dMHSs, such as well-being apps or structured stress management programs, have been considered as helpful for the promotion of patient empowerment and coping strategies.^8–10^ In regards to the treatment of different CMDs with dMHSs, a recent systematic and comprehensive meta-review by Philippe et al. showed that 52% of research on dMHSs has involved the treatment of substance use disorders, 29% focused on mood, anxiety, and traumatic stress disorders, and less than 5% on remaining CMDs.^11^ In general, dMHSs are defined as services that make use of information and communication technology in the field of mental health.^12^ They are considered to be auspicious low-threshold tools or therapy-add-ons because of their flexible modes of delivery, low associated costs, anonymity, and low access barriers since they are location-independent.^13–16^ Other advantages of dMHSs include time flexibility, acceleration of the treatment process and outcome, improved therapy adherence, increased health literacy, simplified contact maintenance, and the management of symptoms of CMDs.^17–19^ In Germany, the “Act to Improve Healthcare Provision through Digitalization and Innovation” (Digital Healthcare Act)—passed in December 2019—allows for the prescription of evidence-based dMHSs (i.e. medical apps) by physicians and psychotherapists, online video consultations, and access to a secure healthcare data network for flexible and location-independent treatment of, for example, CMDs.^20^ The costs for the use of evidence-based dMHSs are reimbursed by statutory health insurances, covering around 73 million citizens.^21^ The German healthcare system is unique worldwide. Other countries, such as France, Belgium or Sweden, are just starting to integrate this concept into their own healthcare systems.^22^

In general, dMHSs have been proven to be effective regarding the prevention and treatment of CMDs, for instance in reducing symptoms of anxiety, depression, stress, eating disorder, or social and academic functioning.^23–27^ However, even though evidence supports the efficacy of many dMHSs concerning an improvement of mental health,^16,28–31^ uptake rates of dMHSs, such as medical apps, remain low.^32,33^ This can be explained by unawareness and skepticism toward dMHSs among patients and healthcare professionals (HCPs) such as psychotherapists and general practitioners, including concerns related to data security, confidentiality, efficacy, impersonality, insufficient information, and low digital health literacy.^8,17,18,34–39^ Accordingly, Gerlinger et al. pointed out that HCPs do not feel well informed about benefits and risks of dMHSs,^8^ while other studies showed that only few of them already have practical experience with dMHSs.^17,40,41^ For a comprehensive dissemination of dMHSs into the healthcare system, it seems logical to educate future HCPs, such as medical and psychology students, for several reasons. First, as a consequence of the Digital Healthcare Act, HCPs will have to deal with manifold questions related to digital health in their practice, which calls for an early acquisition of digital health literacy more than ever before.^20^ This early acquisition also seems important in regards to giving future HCPs the possibility to gain practical experience with dMHS during their studies, which has been shown to be a determinant of dMHSs’ acceptance.^17^ Second, future HCPs will represent the gatekeepers of healthcare delivery because they are the primary source of health information for many patients, thus having a large influence on their attitude formation.^42^ Lastly and most importantly for our research interest, they are potential users of dMHSs themselves since they show high proportions of distress^43,44^ and represent a high-risk group for CMDs.^45^ They report manifold attitudinal barriers to seeking help^46–50^ and still tend to have little knowledge about mental health services.^36^ Attitudinal barriers include the preference to manage problems on one's own, low help-seeking intentions, expected career disadvantages, fear of stigmatization, and skepticism about the efficacy of care.^46–50^

To tackle these concerns and close knowledge gaps, tailored acceptance-facilitating interventions (AFIs) such as multi-component information strategies have been found to be a promising tool in educating individuals about innovative approaches, such as dMHSs.^51–60^ For instance, Hein et al.^61^ could show that physicians’ acceptance of health apps focusing on chronic pain was strengthened by a short educational video providing information about the content of health apps, for example, how they can be used and evidence of recent studies. Credibility and performance expectancy were the strongest predictors of acceptance, followed by skepticism.^61^ Among psychotherapists, Baumeister et al.^52^ found that acceptance of blended therapy might be improvable by AFIs, particularly in subpopulations that were initially rather skeptical such as psychodynamic oriented psychotherapists.

Despite the stated reasons for educating future HCPs on dMHSs and initial positive findings on the usefulness of AFIs in improving the acceptance of dMHSs, there is insufficient research on their needs and preferences regarding how and about what they wish to be informed as potential users of dMHSs.^16,51,53^ Most research has either focused on university students’ preferences regarding the design of dMHSs^62^ or their attitudes toward dMHSs,^63^ but not on how information about dMHSs should be disseminated to targeted recipients to facilitate acceptance. Some studies provided at best few insights into the design of AFIs on dMHSs.^54,55,64–67^ For instance, Apolinário-Hagen et al.^68^ investigated the influence of information with or without varying testimonials but found no change in attitudes toward dMHSs among university students, while a follow-up experiment indicated positive findings on attitudes and acceptance using optimized AFI material with testimonials.^67^ Research on future HCPs as a specific student group is even scarcer. However, this is an essential first step that has not been covered by research yet, which could be one reason for the low uptake rates of dMHSs. Until today, it remains unclear how information strategies on dMHSs should be designed and disseminated to meet the preferences and needs of both uninformed and possibly distressed students that will one day become HCPs. In conclusion, an in-depth understanding on what they would like to be informed about by whom as well as on how they would like to be informed is needed to design effective AFIs on dMHSs for the early acquisition of digital health literacy.

Hence, the purpose of this study was to explore information preferences and needs on dMHSs among medical and psychology students as potential users of dMHS who are at risk to develop CMDs. At the same time, according to the Digital Healthcare Act physicians and psychotherapists are the two groups that will be allowed to prescribe medical apps in the future and thus will have a large influence on the adoption of dMHSs in Germany.^20^ We were interested in (a) exploring design as well as content needs and preferences regarding information on dMHSs and in (b) identifying the most relevant components (i.e. attributes) and their possible levels which together constitute AFIs (i.e. information strategies) on dMHSs. An example for an attribute could be “information source,” while a corresponding attribute level could be “university” or “HCPs,” representing the source where the information on dMHSs for students come from.

## Methods

### Study design

Given the explorative nature of the study, a qualitative design was chosen as a first in-depth analysis. It is most suitable for application in areas where information seems incomplete or not yet attainable through quantitative approaches.^69^ To get a thorough understanding of students’ information preferences and needs related to dMHSs, we conducted semi-structured video-based individual interviews via the software Webex™ by Cisco (Version 41.6.0.19119). The results of this study further help select and specify attributes and attribute levels for information strategies on dMHSs. The ethics committee of the Medical Faculty of the University of Duesseldorf approved the study (study number 2020-972). The study was preregistered at the Open Science Framework on August 11, 2021.^70^

### Sampling

Students who were 18 years and older could participate in the study if they were enrolled at a German university in medicine or psychology (bachelor's or master's program), excluding students from other health-related disciplines that will not be allowed to prescribe medical apps in the future. We followed a convenience sampling strategy, that is, for example, recruiting via social media, personal contacts or flyers and posters at universities. To ensure a wide range of characteristics, we intended to recruit participants from all over Germany. Additionally, our strategy was to recruit specifically for participants with a wide variation of characteristics (e.g. age, gender and federal states) to increase the chances of obtaining differing perspectives. Medical and psychology students who expressed interest to participate in the study were provided with more details of the study and the participant consent form. Participants who did not provide written informed consent were excluded from the study.

### Data collection

Based on research literature on possible attributes of information strategies on dMHSs we developed a preliminary topic guide with eleven themes and possible follow-up questions as well as a short background questionnaire covering questions about demographics, familiarity with and readiness to use dMHSs.^52,55–57,60,64,68,71–75^ Participants who provided written informed consent were asked to complete this questionnaire before the interview. The interview guide was developed by AKS and JAH, discussed in the team (AKS, JAH, AL, PB), and pre-tested by AKS. In total, six test-interviews were performed (*n* = 2 male, *n* *=* 4 female), simulating an interview under realistic context conditions. In addition to conventional pretesting, comprehension probes was used as an element of cognitive pretesting to collect further information about the way participants understand certain questions or terms (e.g. What do you understand by the term *e-mental-health*?).^76^ Results led to small changes in the topic guide and in the background questionnaire.

In the beginning of each interview, the term *digital mental health* as well as examples for dMHSs were introduced. Data were collected by AKS and PB from August to September 2021 until consensus on thematic saturation was achieved, that is, no substantially new content emerged from the interviews.

Online interviews were recorded as audio files with an external recorder. The audio files were transcribed by an external transcription provider and analyzed (verbatim) for content analyses. Interviews were conducted in German and quotes were translated into English by a researcher and professional translator (LG) for this publication. All procedures strictly adhered to the Declaration of Helsinki in the latest version and applicable regulations (e.g. General Data Protection Regulation, Federal Data Protection Act).

### Data analysis

Qualitative content analysis was performed using the MAXQDA 2020 software (VERBI GmbH, Berlin, Germany) based on the approach of Mayring.^77^ According to Mayring, categories can be either formed deductively from theory and then assigned to text passages, or inductively out of the data. Correspondingly, we applied a combination of both inductive as well as deductive coding. During interview transcript coding, main categories (attributes) were formed deductively according to the preliminary topic guide which we developed based on a literature seach..^52,55–57,60,64,68,71–75^ Within these main categories, subcategories (attribute levels) were formed inductively based on the transcripts. PB and AKS independently coded six interview transcripts. These six interviews were chosen based on the distribution of gender, federal state and study program to ensure heterogeneity. Subsequently, the category systems and text samples were compared, and discrepancies were resolved via discussion, which led to the preliminary coding scheme. PB then performed the qualitative analysis of all remaining interview transcripts according to this scheme, which was extended. After completion of the first coding round, the scheme was further reviewed and slightly modified by JAH as principal investigator as well as by AL, who is an experienced researcher in the field of qualitative data analysis.^78–80^ The revised coding scheme was then again applied to all transcripts in a second round of coding. As only small modifications were made during the second coding round, two coding rounds were deemed sufficient. Additional file 1 presents the final coding scheme. The conduction and reporting of findings followed the checklist of consolidated criteria for reporting qualitative research (COREQ)^81^ and further recommendations on formative qualitative research in preference elicitation.^82^ The members of the study team that were engaged in the analysis process had different professional backgrounds (i.e. medicine, psychology, public health, and epidemiology), which should ensure intersubjective transparency, replicability, and discriminatory power of the categories.^83^

## Results

### Sample description

In total, *n* = 21 online interviews were conducted with an average duration of 31.7 minutes (range 16–55 minutes) including *n* = 16 medical students (*n* = 4 male students) and *n* = 5 psychology students (*n* = 1 male student), who participated in the study. Table 1 summarizes the main characteristics of the sample. On average, students were *M* = 25.5 years old (standard deviation [*SD*] = 3.86, range = 20–33) and studied in five different federal states of Germany (North Rhine-Westphalia, Baden-Wurttemberg, Hesse, Bavaria, Rhineland-Palatinate). Among all students, *n* = 11 (*n* = 7 medical students) had already completed at least one educational program, such as surgical technical assistant, nurse or paramedic, or study program, such as a bachelor's degree in molecular medicine or psychology, before being enrolled in their current study program.

**Table 1.** Sample characteristics.

Variables	Mean (Min–Max, SD) or *n*
Students (*n* = 21)	
Interview duration in minutes	31.7 (16–55, 10.3)
Codes linked to interviews	679
Age in years	25.52 (20–33, 3.86)
Female	16
Male	5
Subject: Medicine	16
Subject: Psychology	5
Earlier completed training or studies	11
Location: North Rhine-Westphalia	7
Location: Baden-Wurttemberg	3
Location: Hesse	1
Location: Bavaria	5
Location: Rhineland-Palatinate	5

### Attitudes toward dMHSs

Generally, *n* = 20 of the interviewed students reported positive attitudes and said they were open toward the use of dMHSs as a preventive service if they were offered free of charge by their university. Most mentioned areas of interests were stress management, sleep difficulties, exam nerves, concentration problems, and generally overcoming fears.

Many students reported having little knowledge about or no experience with dMHSs. However, more than half of the interviewed students (*n* = 13) had at least heard about dMHSs, such as apps for meditation or online stress prevention programs, while only *n* = 2 students had heard about medical apps before (i.e. dMHSs on prescription). Furthermore, students expressed that the topic of digital health had barely been mentioned in the course of medical or psychology studies, even though it was perceived as a crucial topic both for personal needs (e.g. during stressful exam periods) as well as in preparation for their future work as HCPs.

Most participants did not favor information strategies on dMHSs for personal use that were designed for medical or psychology students only. Instead, they favored information strategies on dMHSs that generally targeted students’ needs, as they did not perceive any relevant difference between student groups in terms of stress and mental health problems.

### Information sources

**Figure 1.**
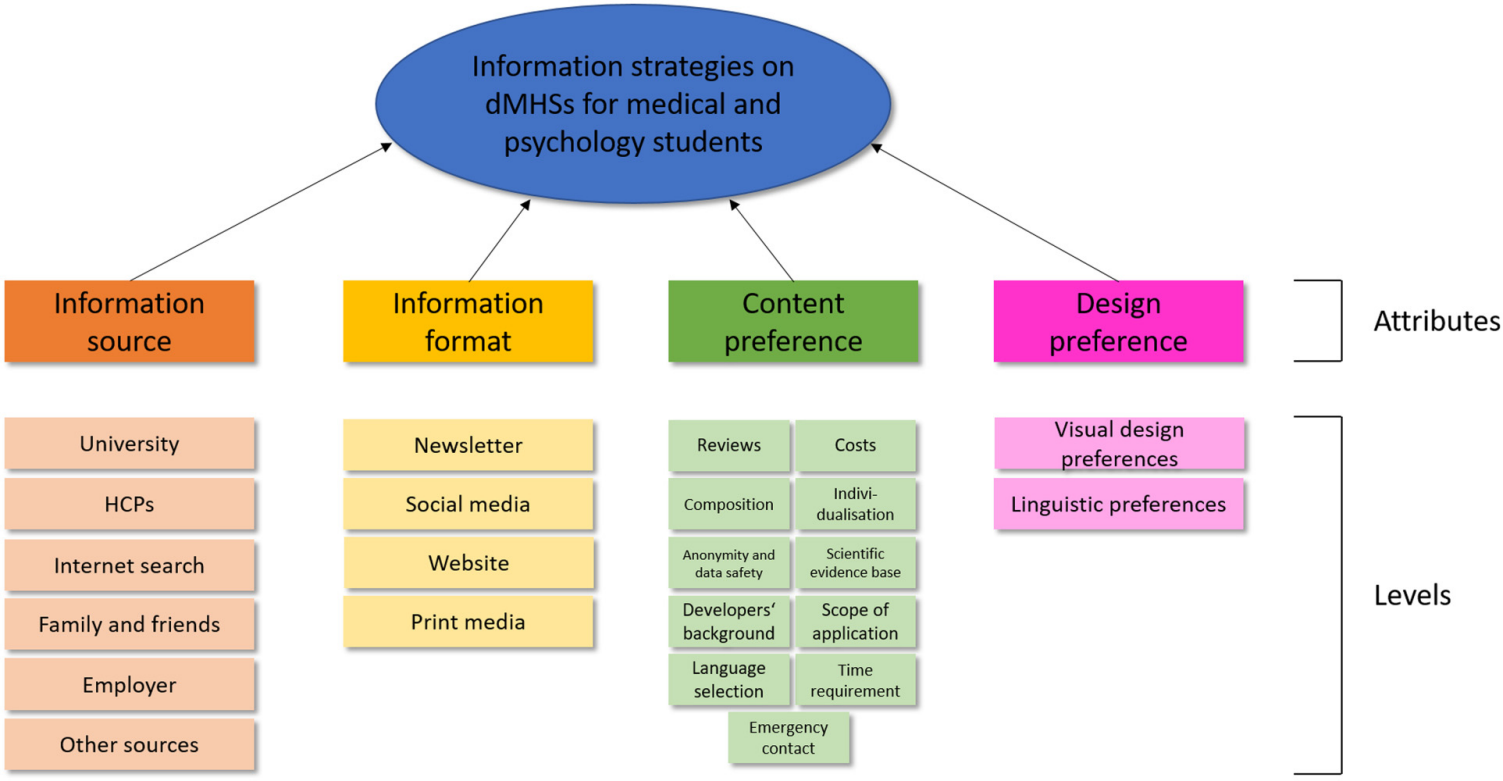
Overview of attributes and levels.

Figure 1 provides an overview over all deductively derived attributes and inductively formed levels of attributes that, in combination, could constitute an information strategy. Our results showed that an information strategy consists of an information source, information format, content preferences, and general design preferences. An example is that medical and psychology students as future HCPs could be informed by their university (information source) via social media (information format) on the scientific evidence base of dMHSs (content preference). This social media post could be written in cheerful, humorous language, designed in light blue and green colors (design preference).

#### University

Students mentioned different ways of how they would like to be informed, but many indicated that, in their opinion, the university is mainly responsible for information provision. Students wished to get a clear overview and recommendations for dMHSs by their university because of the large number of existing dMHSs, such as commercial mental health apps. They said that they missed guidance or counseling at their university, both from the perspective of a potential user of dMHSs as well as with respect to their planned future career as HCP. Additionally, students indicated that they would like to be informed about dMHSs right at the beginning of their studies, as a preventive service, and highlighted the importance of proactive information provision. Many students reported a lack of knowledge of existing psychological support structures available at their university.

“*So, I think it would be useful to talk about this topic in general at the beginning of any study, because stress is such an important issue nowadays, no matter whether it's during your studies or in your job. And I think that everybody, including me, deals with stress in the studies, at least quite often. Which is why this is a topic that needs to be addressed more often in general. And in this context, when talking about it, it would be actually very helpful to immediately provide different services on how it could be implemented.” (373OEJUG)*

Furthermore, some students said that they would like digital health, including dMHSs, to be introduced as an integral part of the education of future HCPs. When asked for the preferred form of information transfer, many students favored interactive forms such as seminars as part of an elective subject.

“*There are different ways of exchanging information with each other, for example presentations that can be given in turn. You could maybe do a course, a minor subject, where everyone gives a small presentation on a subtopic of e-mental-health, like five or ten minutes, that would be enough, and you’d have heard about it at least, so that you’re not completely on your own.” (POX4QTL2)*

However, some of the medical students expressed concerns about providing information on dMHSs only in an elective subject since they feared that information might get lost this way. These concerns include a limited number of electives that students can choose, restricted access to elective subjects as well as too little information in the description of each elective.

“*I think corresponding elective subjects would actually be a good idea, but I also have to say that you don’t really get much information on some of these subjects (…), well some of them have a small description, (…) but I don’t know if some students decided not to take it, simply because they wouldn’t have enough information (…) and you can also only choose a limited number of elective subjects and some of the subjects have a limited number of participants, so ultimately, the majority of students wouldn’t take part probably. Exactly, so there are special services, but access is also limited.” (W0366MAW)*

Instead, some students expressed the idea of digital health being introduced as an integral subject of their studies or as a compulsory seminar, for example, for all freshmen. Furthermore, students reported several other possibilities to get informed by their universities including services from the student representatives, the student services center, student working groups or the deanship.

#### Healthcare professionals

Students mentioned HCPs as another trustworthy source of information regarding dMHSs because they were often seen as primary and trustworthy source since they provide their patients with reliable information. However, some students stated they would feel uncomfortable asking HCPs for advice as they would often seem annoyed or stressed. Additionally, some students showed general low help-seeking intentions because of expected career disadvantages, which still appears to be a widespread reason for not seeking support at HCPs or health insurances.

“*Health insurance might also be an option, but somehow…well, it could be said—and I have also noticed this with my fellow students—that they were having some problems in their studies, and they were like: Alright, I won’t go to a doctor or a psychologist now, because I know that I can’t practice some professions if I receive certain diagnoses. And I don’t know in what way they are worried in advance maybe, so that they might hesitate to use the services. So, I think I’d prefer the most anonymous way, like using google or the internet.” (57VOPFC7)*

Some students reported that they could imagine that the exchange with other HCPs as information source for medical innovations such as dMHSs might become more important once they are in that role themselves.

#### Internet search

If interviewees already had experience with dMHSs, they most often mentioned own internet search as their initial information source. In accordance, when asked how they would now search for support in stressful phases, for example, during exam periods, almost all students replied they would “*use google*” *(DVHQI4ZS)* and see what they could find on the internet.

Some students stated they found specific dMHSs through app store research or personalized advertisement on social media.

“*And well, if you do some research on the topic you mysteriously get corresponding ads, on Instagram for example. And I think I found one of them myself, I had searched for it and clicked it and the other one was suggested to me, yeah, that's more or less how it was.” (B3ACVZZ6)*

However, most students said that they would prefer to be informed about dMHSs by their university or HCPs instead of doing internet research themselves because they thought that “*the risk of drawing a blank is way too high” (MHMEUCIZ)*.

#### Family and friends

Some students also reported that if they had already used services for their mental health, they got their information about these dMHSs from friends or family members. Since significant others are usually perceived as a trustworthy information source, some students described word-of-mouth as a good option to get informed. In general, they said that they would rely on their family or fellow students and friends when it comes to mental health because they might either experience similar stressors or would only recommend services that they also benefited from in the past.

“*Well, I would say close family members and friends, of course, simply because they are people that I know. And I know that they share their personal opinions with me, without any hidden agenda. This is actually always the best thing, if they have no personal benefit, they are being honest. They tend to give me their honest opinion. And therefore, I think that I’d immediately do so I’d say.” (373OEJUG)*

#### Employer in healthcare

Another idea to inform future HCPs about dMHSs was through the employer. Especially for medical students, who must complete several internships at, for example, clinics, during their studies, it was mentioned that they would appreciate being informed about health promotion services by their employers as they should also have an interest in their staff's overall health.

“*I think it would be really cool if my employer did that. Simply because they should also be interested in me doing well in the job for a longer time. And especially because—well, I work at a university hospital myself; it's a huge organization and they also have far more resources that are used anyway, for, I don’t know, all sorts of studies, PR. So, I’d be really happy about more services for the employees, I would say.” (OEFFMG40)*

#### Other sources

Finally, some students mentioned additional information sources that do not fall into the deductively defined main categories. These include public bodies, such as the federal ministry of health or the federal ministry of education, and research but also TV advertisement. Regarding public bodies, students mentioned that they would assess them as trustworthy sources when it comes to mental health recommendations, as opposed to websites of dMHSs where these services are to be sold. Interestingly, one student did not see information from dMHSs’ producers as problematic, but mentioned that, for example, TV advertisement of dMHSs could indeed be another valuable source of information because many persons could be reached.

“*I could also imagine advertisement on television. From my point of view, there should be way more advertising for health services. You should just try to reach as many people as possible. Not everyone has a cell phone, especially elder people.” (KB4I9VSV)*

### Information format

#### Newsletter

Students imagined several different ways of how to get informed about dMHSs. One possibility mentioned was sending a newsletter to all students, for example, from the student services center, the deanship, or by health insurances. However, the usefulness of these newsletters was also discussed.

“*It's always quite difficult to really reach all students. Sending an e-mail would probably be the easiest way, just sending it via mailing list. But honestly, I don’t know if that's a good idea, because e-mails that come via mailing lists, for example from the deanship or something like that, are often skipped, I think. You just skim them, and then you notice: Okay, it's nothing important, and it goes straight into the trash or storage. It might work better with a personal form of address or some advertising by the lecturers or in seminar groups; places where you are in smaller groups, but I’d still say that you reach the most students via e-mail and effectively also those who are interested in it after all and who notice it and be like ‘Oh, this does even exist?’ (…) Everyone who is not interested or who doesn’t need it can still delete the e-mail. So yes, I think this is still the most effective way.” (TNN4A5ZZ)*

#### Social media

Regarding the use of social media in information strategies, students had dissimilar opinions. Some stated they benefitted from personalized advertisement on, for example, Instagram, whereas others argued that they were not sure whether information on dMHSs retrieved from social media platforms was trustworthy. However, if the information source is assessed as reliable (i.e. the institution behind the social media profile, such as the student services center), presented information is more easily accepted. Additionally, students mentioned Facebook student groups as another option to stay updated. Many reported that they had heard from the possibility to participate in this interview-study in a Facebook group from their course of studies and that they could imagine being informed about dMHSs in these groups, too, for instance, by student council groups. Podcasts were also mentioned as a possible way to retrieve information on dMHSs, especially because one can be notified about possible updates such as new dMHSs. This way, students reported that social media could be an effective and low-threshold way to reach those in need.

“*The university also has two Instagram accounts now, for example. So, generally speaking, it would also be possible via social media. (…) Therefore, yes, trustworthiness would definitely increase for me if it were a university recommendation.” (W0366MAW)*

#### Website

Lastly, some students wished for a website, for example, hosted by the university or health insurances, where all information on dMHSs and other services for mental health could be listed and shortly explained to get an overview. In their point of view, this would reduce uncertainty and save time.

“*Well, I think it would be good if there was some kind of platform that has all the information. So, that you could list different apps and additional online therapists or something like that, that you’d have at least a phone number or a contact person.” (WVRROB3C)*

#### Print media

On the other hand, two students stated that they would prefer print media, such as flyers, brochures or scientific literature from the university library, over digital platforms so that they would have something tangible at hand.

“*I always think that it's nice to have something tangible at hand, which is why I thought of flyers first. Something that you can also pass on, but of course, that's true for an e-mail or an info page, as well. I just prefer having things in my hand.” (TNN4A5ZZ)*

### Content preferences

#### Reviews and recommendations

For many students, online reviews by other users seemed to be important information on dMHSs that would influence their usage intention because it gave them an initial indication of whether dMHSs were worth engaging with. For instance, when searching for a mental health app targeting a specific disease pattern such as depression, students appreciated it when users reflected on whether this app was helpful or not. Additionally, students wanted to know whether dMHSs were intuitive, technically well-established and self-explanatory, for example, through self-reports by other users. When reading online reviews, many students seemed to specifically look for detailed, supposedly honest opinions.

“*You usually notice if they are written honestly and then you can really work with them. That is, if you have concrete and positive feedbacks instead of ‘Oh yes, I feel totally great now’. Instead of this general feedback you could mention precisely what you liked about it. So, I mean concrete feedback and not just a good rating.” (G58596A1)*

However, most students also expressed their skepticism about online reviews and even mentioned that it would scare them off because they did not know whether they could take them seriously. Additionally, a few mentioned that they regarded online reviews as not meaningful because they perceived mental health as a topic that is too individual and thus cannot be transferred to others.

“*Of course, I’m really happy if someone writes: ‘It really helped me a lot, I feel much better’. But in the end, this can’t necessarily be related to your personal usage. Unless it's about technical issues (…). But for me, this was no exclusion criterion nor was it a selection criterion. If the ratings are great, it doesn’t necessarily mean that it's great for me, too.” (0SCEPLP9)*

A lot of interviewed students stated that they would be more willing to use dMHSs that were previously tested and approved by friends, students, or university lecturers because students had difficulties to decide as “*there are 1000 offers, and most of them cost a little bit” (B3ACVZZ6).* Many students favored dMHSs that were recommended by central institutions such as universities or health insurances as well as by HCPs. Thus, including those recommendations in information strategies on dMHSs seems critical.

“*Well, with a psychotherapist, you simply know: Okay, she has experience with this. Others have also tried it before. So, there is some personal experience involved.” (WVRROB3C)*

“*Certification is also very appealing to me. (…) Let's say, for example, there is an app that should help with the treatment of depression, and I would either be in the role of the therapist or also in the role of the patient. For me, it would be extremely helpful if it simply said: accepted by the German Psychologists’ Association or found to be good.” (KB4I9VSV)*

#### Costs information

The importance of information on costs was highlighted by most students. No or low costs seemed to be a decisive factor for the uptake of dMHSs as students often do not earn a lot of money. Thus, this information seems essential for information strategies.

“*Well, I don’t really earn much as a student and I’d really like to save some money or get special offers, a discount for students or something like that.” (POX4QTL2)*

In general, students agreed that information strategies on dMHSs needed to include information on costs because high monetary expenses were seen as a barrier for usage. Students agreed that if dMHSs were not free of charge, they would like to be informed whether trial subscriptions were offered because they wished to have the opportunity of getting to know the service or app before paying for it.

“*And I think it would be important that it's free of charge. I think, a lot of people are put off if there are any costs for potential users. (…) Or that there is, I don’t know, a time period where you can try it out for free. (…) I mean, of course, all of this has to be paid for, without a doubt. But then you should perhaps have the opportunity to test it first. And then, you can buy it if you are convinced of it.” (DVHQI4ZS)*

#### Anonymity and data safety

Students held varying opinions about data safety, that is, whether they wanted to be informed about this topic as part of targeted information strategies on dMHSs greatly differed. For some students, data safety was essential, especially in the context of a sensitive issue like mental health. Many reported that they would not like to use dMHSs that required entering personal data, because talking about mental health issues seems to be stigmatized among future HCPs. Thus, this information should be presented before using dMHSs due to the importance of privacy and discretion.

“I wouldn’t really want to share personal details on an app like this. (…) I mean, I don’t really know how you could trace back that I am using the app as a person concerned. Because it just occurred to me that if people find out that I have this app, then there is a certain stigma to it, (…) so that you wouldn’t really want to mention that you get psychological help. This always makes me really sad. (…) But yes, I’d say data you put online should be reduced to the minimum.” (*MHMEUCIZ)*

Some students stated that information on whether data is treated in accordance with the General Data Protection Regulation (GDPR) or a GDPR certificate would be sufficient to be perceived as trustworthy as realistically no one reads the general terms and conditions. Others reported that they did not necessarily need to have information on data safety when educated on dMHSs because their data was already “out there,” but pointed out that anonymity would play a crucial role for usage.

“*I must say data safety (…) has not been an issue I’ve been worried about, maybe because I know that you can’t really do much with the data. (…) I’ll be there, and I might be talking about my problems, it's anonymized maybe. And even if that's not the case, it falls under some form of data protection law anyway. (…) I think many people are more willing to pour their hearts out and talk about their problems, for example in an online forum, if they are anonymous, because, let's face it, nobody wants to have their name and picture there and talk about crying all night long because of an exam. So, I can imagine that this is somehow liberating and that it motivates people to exchange experiences. So that you can find other people who are going through similar things.” (HNIQK3ZD)*

#### Individualization

Some students wished to be informed about whether a service or mental health app offers individualization options, for example, by using a questionnaire to diagnose symptoms at the beginning and then customize the content of dMHSs accordingly to their needs. Similarly, they would like to know whether dMHSs are flexible in terms of exercises and their duration, so that they could choose tasks according to the time available.

“*Or, for instance, that you have various exercises that you can choose from. How much time do you have right now? What would you like to do? And then, it should be possible to choose between five, ten, or twenty minutes. I think I would like something like that.” (KB4I9VSV)*

#### Composition

Students said that they wanted to be informed about the content and structure of dMHSs, for example, how many courses a program consisted of or whether one could choose between different subscription options, including a test subscription that offers an insight into the program. They also wanted to know if dMHSs offered a wide range of content to quickly determine whether they fitted their individual needs.

“*I think it would be nice to get a clear insight at first, to see what the program includes, to determine its strengths, so to speak. I mean, there are different kinds of relaxation techniques or possibilities. It would be good to have an overview, because some things just don’t suit you.” (G58596A1)*

Furthermore, interviewees valued dMHSs that were diversified and did not quickly become boring because they favored services that covered different interests. Thus, this was an additional point that they wanted to be informed about.

“*I would really appreciate it to see that there are, like four different aspects. It's not always the same, it varies and maybe I don’t have to answer each aspect every day. I think it would be quite interesting to see that a lot of aspects are covered, that it's not just ‘are you sad?’, ‘are you happy?’ all the time. Which means that I’d be appealed by this distinctive character, (…) I need to see that many aspects are covered.” (MHMEUCIZ)*

#### Scientific evidence base

Students considered it crucial to be informed about the scientific evidence base of dMHSs. They wanted to know whether there was sufficient empirical support for dMHSs or whether, for example, mental health apps had been proven effective and efficient in trials because otherwise they would wonder why they should even use them. Furthermore, some students stated that they would like to read some information about scientific studies on dMHSs.

“This means that I would find it great if you could somehow retrace the following: Okay, how many participants have tested it and what are the results and maybe also, I don’t know, is any S3-guidline included? For me, a certain evidence level would be important. Because otherwise, I could also go to someone who holds a compass over my chest or something like that. But yes, it would be nice to know that work was put into it to examine it, and this would make it more appealing in my opinion. (…) For me, it's actually enough to see: Okay, it has been examined by a research group. They have good results, six university hospitals are using it, and then I’d definitely be convinced of it.” (MHMEUCIZ)

#### Background of developers

The scientific evidence base was often mentioned in combination with the background of dMHSs’ developers, meaning their professional expertise. Informing students on whether the team of developers included, for example, psychologists and specialized physicians seemed to increase integrity.

“*So, yes, I think it's always good when there is a certain professional background somehow. For example, that (…) psychologists have been involved in the development of it maybe. (…) I think this enhances your trust in the app. Because you feel that it's really useful and even though it won’t replace therapy, (…) you can start helping yourself with it.*” *(5DV5XVFM)*

Additionally, informing students on who developed dMHSs was considered to expand long-term attachment.

“*I also think it's nice to know who is behind it, so who has developed it and why. I think that's especially important, because it binds you to the whole matter in a different way.” (OEFFMG40)*

#### Scope of application

Interviewed students wished to be informed about the intended target group, such as seniors, employees or students, because they could then evaluate whether a specific service could be applied to them. They also stated that they would like to be informed about the intended purpose of dMHSs, that is, for which problems and diseases dMHSs were specifically developed. For instance, if students explicitly wanted to learn to cope better with exam nerves, they wanted to immediately see whether a program was developed with this intention.

“*Well, if the meaning, the purpose or targeted diseases of the app are adequately formulated in advance, open and honest, or (…) what you’d like to accomplish with it, then it's probably more appealing to everyone—including me—compared to when nothing is really expressed clearly, and you’d practically have to try to get along with it, and see if you can find anything at all. Because this would actually set me back a step right away.” (OEFFMG40)*

#### Emergency contact

In case of psychological emergencies, students wished for dMHSs that could provide suitable contacts as well as quick, reliable information in order to help patients in acute need. In this case, anonymity would lose importance.

“*Also, to what extent you are forwarded to non-digital locations, let's say, in case of acute need. This should be present as well. People should not be pushed into a digital service, and don’t come out of it in that sense. I think that's the most important aspect.” (0SCEPLP9)*

#### Time requirement

Additionally, students stated that they would dislike time-consuming dMHSs as they already have a stressful everyday life as a student. For this reason, they wished to be informed about the recommended daily effort that was required to achieve results before usage.

“*And maybe also the time limit (…). Some (…) also advertise with something like: ‘Seven minutes per day and the day is less stressed.’ This would also be important for me as well (…). I don’t want to install the app and then enter information for over an hour. Maybe you weren’t even prepared for this, because you don’t have enough time.” (AT9OOOCZ)*

#### Language selection

Furthermore, one student mentioned that she once unintentionally downloaded a mental health app that was only available in English, which increased the threshold to use it. Another student stated that, especially as future HCPs, it would be desirable to be informed about whether dMHSs were available in different languages so they could be recommended to patients with varying mother tongues. In general, talking about emotions and struggles seems to be easier using dMHSs in the native language.

“*Well, I just feel more comfortable there. It's not like I don’t understand it or something like that, but I can just kind of let go.” (B3ACVZZ6)*

### Design preferences

#### Visual design preferences

Because of limited time resources, most students preferred images or short videos in combination with brief, explanatory text in terms of information strategies. For instance, when sending a newsletter to students, interviewees preferred some facts about dMHSs, such as costs, recommendations, and scope of application, and only a few images or videos about the content and structure of the app.

“*I’d say I really like short and concise sentences. (…) When I’m not feeling well mentally or when I’m stressed, and I’m looking for such a service, I don’t want to have to go through the cognitive effort of reading long texts. (…) Short, concise sentences that stay in mind, a bit like a mantra maybe. Definitely pictures. Animations would be nice as well.” (HNIQK3ZD)*

On the other hand, some students preferred texts over videos. They argued that students were often in public locations, such as cafés, where videos were harder to watch:

“*I can also imagine videos, especially in the context of explaining how things work. For example, in the app, you can show people how to use everything. And here you have the different functions. (…) But I think that text is one of the clearest things. Because if you are out, for example, and you want to get some information, you wouldn’t watch a video. Well, I think that many people do some research when they are not at home. Sometimes, they are in a café, at university, they’re working. And I always think that videos are a bit inconvenient in that case.” (KB4I9VSV)*

When dMHSs provide personal contact with HCPs, some students had the idea of portraying the experts to make dMHSs more appealing and credible.

“*It has a different effect if there's a person on it, compared to, I don’t know, if you have a picture of some landscape or something else, or no picture at all. But I think it would really make a difference, for me personally at least, because it's more appealing.” (5DV5XVFM)*

A few other students had the idea of a modular composition of information, giving interested recipients the opportunity to dig deeper into specific themes.

“*Actually, I like to have a brief summary of the most important information and then, there is additional information in the background, so to speak. For example, that you have the possibility of reading more on an individual issue if you want to do so. But that everything is very compact at first glance, especially if you want to compare things.” (KB4I9VSV)*

Congruently, students favored information material in light, subtle colors, such as light green or light blue, because they seem to be associated with the healthcare sector and general professionalism.

#### Linguistic preferences

Regarding linguistic preferences, students had different opinions. Some preferred scientific language and the focus on facts, others would rather go with cheerful, humorous language because addressed topics are already serious enough.

“*Yes, I think you catch more people with casual and humorous behaviour. If you really start casually and with humor. This means I wouldn’t read it probably, if it were just facts. If I want to read about facts, (…) I open a book. And I just think that you should somehow also see things in a relaxed manner.” (GFUI3FCB)*

## Discussion

The aim of the present study was 1) to explore medical and psychology students’ needs and preferences regarding information on dMHSs and 2) to identify attributes and attribute levels that help to design acceptance-facilitating information strategies on dMHSs. Despite future HCPs’ important role as gatekeepers of healthcare innovations, such as dMHSs,^42^ and their high risk to develop CMDs themselves,^45^ only little is known about their preferences and needs on dMHSs.^84^ Our results may help fill this research gap.

Overall, almost all interviewed medical and psychology students reported to be open toward the use of dMHSs as a preventive service if they were offered free of charge by their university. However, participants still had little experience with dMHSs. Even though the German Federal Institute for Drugs and Medical Devices already included the first medical apps into the prescription index in October 2020, only a few participants had heard about medical apps before.^85^ This result is in line with prior research, showing that future HCPs report little knowledge about and experience with such apps.^36,86^ Interestingly, 14 out of 34 (retrieved on December 23, 2022) approved medical apps address the management of mental health problems and some of them are even tailored at young adults.^87^ However, the lack of information on dMHSs seems to have led to a lack of awareness of these offers until today. Accordingly, results of a recent survey by the Fraunhofer Center for International Management and Knowledge Economy IMW showed that even practicing HCPs seem to have low digital health literacy, potentially explaining low uptake rates.^41^ At the same time, knowledge about dMHSs was perceived as important by interviewees both for personal needs as well as in preparation for their role as HCPs. Again, this highlights the need for structured education programs.^17^ Additionally, interviewed students outlined the importance of proactive and preventive information provision because they often experience stress from the beginning of their studies, which is also in line with prior research.^43,47,88–90^

As possible sources regarding information provision on dMHSs for medical and psychology students, their university, HCPs and health insurances, personal internet search, family and friends, employers as well as other sources such as federal ministries or TV advertisement were mentioned. In accordance with other studies, participants sometimes preferred to talk to friends or relatives instead of consulting HCPs because they feared stigmatization and embarrassment.^34,46,47,50^ However, interviewees favored to be informed by their universities on dMHSs because they wished for a source that can give clear recommendations and guidance for the selection of evidence-based dMHSs. This is in line with previous research outlining that users often feel overwhelmed by the large amount of mental health apps on the market^91^ Similar to the results of Dederichs et al.,^62^ students appear to be more willing to use dMHSs recommended and provided by their universities. Regehr et al.^92^ also see the duty to inform about mental health services on the part of the universities. Due to significant levels of stress in students, they concluded that universities must employ preventative interventions to reach more students. Generally, there seems to be a lack of knowledge of existing support structures available at universities, even though some efforts have already been made by universities to alleviate mental health problems in students. For instance, there is an increasing number of services offered by student services centers at German universities, which could provide help. However, in accordance with our results, they do not seem to reach students in need.^79,93^ Confirmatory, Liu et al.^94^ also concluded that universities need to make more effort to develop strategies to inform those students about the prevention, detection and treatment of students’ mental health problems. However, the lack of a clear allocation of responsibilities regarding the management of these strategies on dMHSs for students’ mental health might still be a potential barrier. It needs to be discussed who is in charge of informing students in order to increase the awareness of the low-threshold, flexible and anonymous services for individuals who fear stigmatization. Our results show that especially for medical and psychology students, the student representatives, the student services center, student working groups or the deanship are regarded as possible information sources.

Regarding preferred ways of how to receive information on dMHSs as potential users, interviewed students mentioned print media, such as flyers, brochures, or scientific literature from the university library, as well as several digital media channels, such as social media (e.g. Facebook or Instagram), websites, and newsletters. Even though social media has been used to survey and educate hard-to-reach populations, such as medical students,^95,96^ to our knowledge there has been no study on how social media campaigns might influence the uptake of dMHSs among students. Our results indicate that, for example, targeted Instagram or Spotify formats on dMHSs, including our identified content preferences (e.g. scientific evidence base of dMHSs, developers’ background, etc.) to regularly inform student populations, might be a promising tool since the majority of students have social media accounts. Furthermore, our results indicate that a website including all relevant and verified information on dMHSs targeted to the needs of psychology and medical students might be beneficial. The German Digital Health Association (German “Spitzenverband Digitale Gesundheitsversorgung”) already hosts a website specifically on medical apps, so a similar tool already exists for practicing HCPs in Germany.

In the university setting, students additionally favored interactive lectures, such as seminars, as information format. Furthermore, students stated that they would prefer the topic of digital health to be an integral part of their study program, either in the form of elective or compulsory subjects. According to Mendes-Santos et al.^97^ the absence of such structured education on dMHSs might be one factor inhibiting digital health implementation at the moment. In Germany, some efforts have been made to change this state of the art with the new version of the National Competence Based Catalogues of Learning Objectives for Medical Education (German “NKLM 2.0”). The NLKM 2.0 is a revised qualification framework for medical students that comprehensively prepares students for their everyday work as physicians with many competence-oriented learning objectives. It now also includes digital health literacy as one of the overarching competencies, which will be part of the mandatory core curriculum in medical studies starting in 2025.^98^ However, the interviewed participants did not expect that respective courses will be about managing one's own health and educating students about dMHSs for self-management. Our results emphasize that there must be a focus on the aspects of help for self-help if one wishes to meet the needs of psychology and medical students in Germany.

Furthermore, participants gave detailed answers for content preferences regarding information strategies on dMHSs. Interviewed students wished to be informed about other users’ experiences with dMHSs, costs, anonymity and data safety, individualization possibilities, content and structure of dMHSs, their scientific evidence base and scope of application, emergency possibilities, time requirement of usage as well as possible language selection. Similar facilitators and barriers to the usage of dMHSs were identified in previous studies.^62,99–101^ In accordance with Dederichs et al.,^62^ information on costs of dMHSs seemed to be especially important because high costs would hinder usage. While Apolinário-Hagen et al.^68^ found no meaningful influence of testimonials on attitudes toward dMHSs, recommendations and reviews by other groups such as users or HCPs were also perceived as essential for information strategies on dMHSs by participants. Those testimonials seemed to be more convincing if they were written in more detail and included strategies to promote the sourcés similarity to the recipients, their expertise and credibility.^102,103^ Interestingly, many interviewed students mentioned that they would appreciate information on the professional background of those who developed dMHSs, which does not seem to have been of special importance in previous research. As we interviewed future HCPs, they might potentially put a stronger emphasis on this aspect because knowing who developed such dMHSs (e.g. other psychologists or medical experts) seems to increase integrity. Further research should investigate whether including this information in information strategies on dMHSs specifically targeted at psychology and medical students might have an influence on the uptake.

Regarding questions on how information strategies on dMHSs should be visually and linguistically designed, students had different opinions, but, in general, content and information source seemed to be of greater importance for participants. Some students preferred short videos that explained dMHSs, others favored text passages with respective images or wished for interactive workshops where students can directly test dMHSs. In general, most students preferred short information including some facts about the dMHSs, such as costs and scope of application. Apolinário-Hagen et al.^54^ found similar results, showing that there might be a positive association between the provision of general facts about dMHSs and attitudes as well as behavioral intentions to future use of such services. Lastly, some interviewed students preferred scientific language and facts, others favored cheerful, humorous language. In order to design different information strategies on dMHSs that fit the varying needs and preferences, it seems essential to determine the relative importance of each of the identified attributes and to identify segments of medical and psychology students based on their shared preferences. To do so, there are possibilities to use research designs that allow for incremental value of different information components,^60^ such as discrete choice experiments (DCEs).

### Limitations

While this study contributed to the understanding of information preferences and needs on dMHSs among future HCPs, it also has some limitations. First, due to anonymity reasons, we did not ask which semester study participants were in. It could be possible that attitudes as well as preferences and needs might change in the course of the studies, considering that students in different years might face different barriers. A further limitation of our study is the potential selection bias in recruiting participants. Possibly, only those students that are interested in digital health might have participated in our study, as we have advertised our study with the question “Interest in e-mental-health?” Thus, the information need for dMHSs might be slightly overrated. Due to the qualitative nature of the study, results are not representative for the entire population of psychology and medical students.. Additionally, the results cannot be transferred to other countries, as study programs might differ across nations and the prescription of medical apps in the German healthcare system is yet unique worldwide. Furthermore, we recruited significantly fewer psychology students (*n* = 24%) than medical students. Even though we could not determine differences in needs and preferences between both groups of students, psychology students were underrepresented. Psychology students as important future HCPs have barely been included in research on the prevalence of CMDs and even less concerning dMHSs’ preferences, thus future research should take their perspective into account in more detail. Moreover, AKS and PB gave a short introduction on dMHSs in the beginning of each online interview, which might have influenced participants’ answers. However, we tried to consider this bias by remaining as neutral as possible during the online interviews and by asking open questions. Nevertheless, it is possible that we still elicited some bias that we are not aware of. In addition, we did not have the resources to return to our participants to check for the accuracy of our observations (i.e. member checking) and to thereby increase our study's credibility. Lastly, although qualitative content analysis is a well-suited approach for application in areas where information seems incomplete or not yet attainable through quantitative approaches, it may be possible that individual quotes and opinions lose meaning during formation of categories and subcategories when reducing the data material.^77^ A further limitation might be the fact that one coder fully performed the qualitative analysis. However, a second independent coder was involved in the formation of the coding scheme. After completion of the first coding round, the scheme was further reviewed and slightly modified by two other independent coders. All coders then approved the final coding scheme. Coding by one author was therefore perceived to be sufficient.

### Implications for practice and future research

The results give first insights into information strategies on how dMHSs should be designed to meet the preferences and needs of both uninformed and possibly distressed students who will become HCPs. Through AFIs in the form of recipient-targeted information strategies on dMHSs, barriers such as low digital health literacy, information overload or concerns about the efficacy and safety of dMHSs could be overcome.^37^ Our results are specifically helpful for, for example, student services centers as they give recommendations on how students in need could be reached and how they could be strategically informed about dMHSs, especially when the treatment demand exceeds their resources.^93,104^ For instance, the student council could inform medical and psychology students about dMHSs by designing a social media post (e.g. for Instagram) with a short video explaining data safety, scientific evidence base and application scope of a specific mental health app for exam anxiety, using light blue or green colors. Alternatively, the student services center could send a newsletter with similar information on dMHSs to all students during the freshman week or shortly before the exam periods starts.

**Figure 2.**
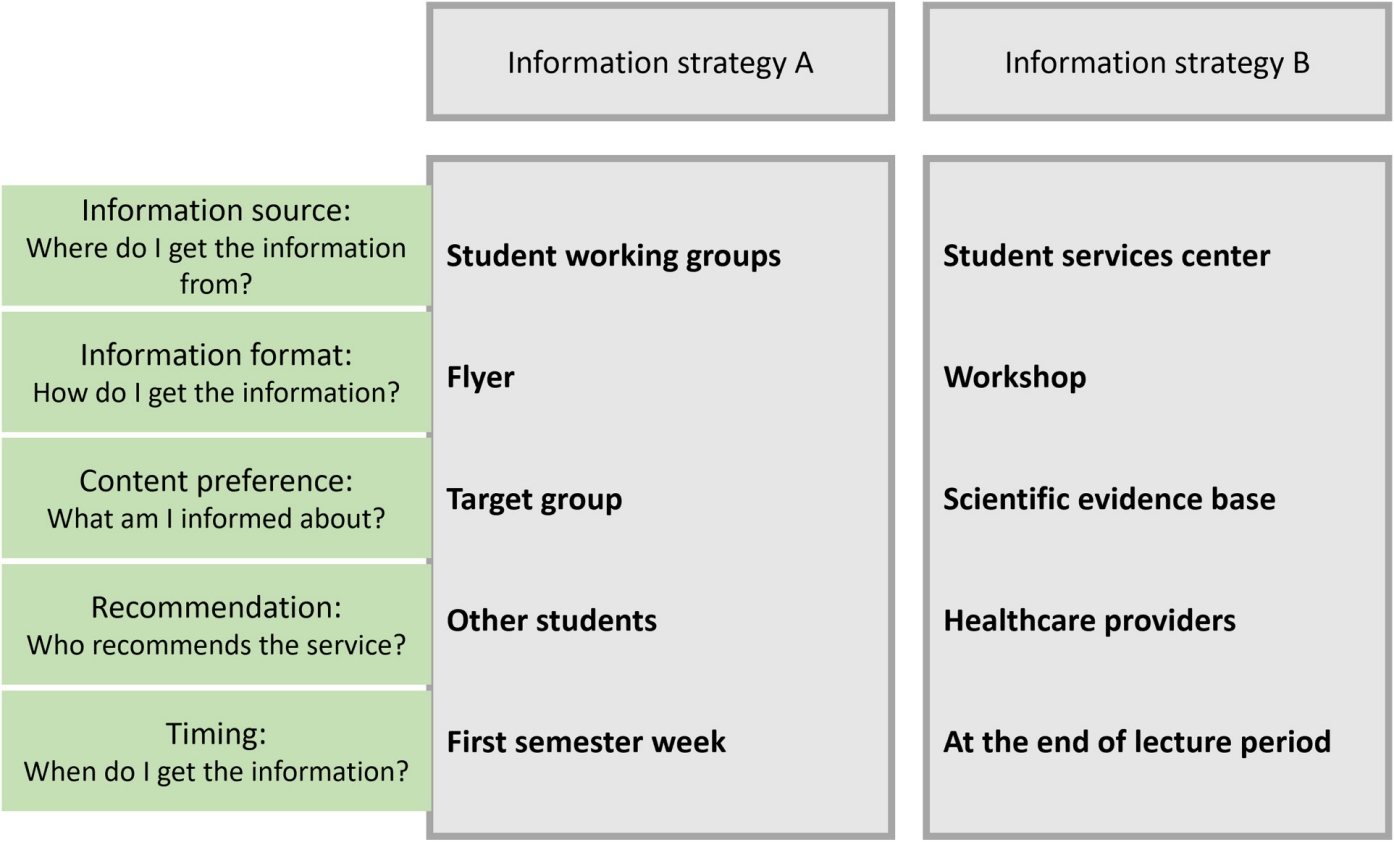
Example of information strategies with varying attribute levels.

Knowing which attributes of information strategies on dMHSs are preferred by medical and psychology students can further help policy makers, mental HCPs as well as product developers to understand why students still hesitate to use dMHSs. Hence, promoting the awareness of dMHSs may be the first step to their adoption based on informed decisions. However, it is still unknown which attributes are most important and how these attribute levels should be combined to constitute effective AFIs. Thus, to increase the implementation of dMHSs and to give clear guidelines, further research mimicking context-sensitive real-world decision scenarios with a representative sample of medical and psychological students is needed.^86^ The focus should be on the systematic variations of the identified attribute levels, as exemplified in Figure 2.^59^ As students stated that they wished to be informed about the topic by their universities, further research should focus on information strategies implemented in the university setting. To identify which components are preferred by medical and psychology students in comparison to others, Ebert et al.^60^ proposed designs that allow incremental value of different intervention components. A DCE format allows for such personalized AFIs entailing a choice between hypothetical information strategies on dMHSs. DCEs offer an empirically grounded methodology to identify important components of information strategies on dMHSs, by modeling the preference strength for a variety of attributes and attribute levels.^74,75^ This would make dMHSs information strategies more tangible to participants compared to conventional survey techniques that do not look for possible trade-offs. Our results can be used for the conceptual development of such DCEs.

## Conclusion

We focused on how medical and psychology students as future HCPs would like to be informed about dMHSs for two main reasons. First, medical and psychology students are potential users of dMHSs since they are confronted with high proportions of stress during their studies. Second, the Digital Healthcare Act in Germany has started to shape the professional routines of future HCPs, which calls for an early acquisition of digital health literacy as they are the gatekeepers for the use of dMHSs. Thus, our aim was to explore their information preferences and needs to design multi-component information strategies on dMHSs as AFIs. We identified various information sources (e.g. university, HCPs), information formats (e.g. newsletter, social media) and content preferences (e.g. reviews, costs) as possible components of such targeted information strategies. Informing medical and psychology students could increase awareness and overcome barriers to the broad dissemination of dMHSs such as skepticism and information overload. Future research should focus on the systematic variations of these components, for instance in a DCE.

## Supplemental Material

sj-docx-1-dhj-10.1177_20552076231173568 - Supplemental material for Investigating information needs and preferences regarding digital mental health services among medical and psychology students in Germany: A qualitative study Click here for additional data file. Supplemental material, sj-docx-1-dhj-10.1177_20552076231173568 for Investigating information needs and preferences regarding digital mental health services among medical and psychology students in Germany: A qualitative study by Pia Braun, Ann-Kathrin Schwientek, Peter Angerer, Lisa Guthardt, Andrea Icks, Adrian Loerbroks and Jennifer Apolinário-Hagen in DIGITAL HEALTH
