# Supplementary material for: Investigating information needs and preferences regarding digital mental health services among medical and psychology students in Germany: A qualitative study
Source: Digit Health. 2023 May 25;9:20552076231173568. doi: 10.1177/20552076231173568 (PMC10226173; doi:10.1177/20552076231173568)
Supplement: sj-docx-1-dhj-10.1177_20552076231173568 - Supplemental material for Investigating information needs and preferences regarding digital mental health services among medical and psychology students in Germany: A qualitative study [file sj-docx-1-dhj-10.1177_20552076231173568.docx]

**Topic guide for an online semi-structured interview**

**Date:**

**Code:**

*** = Optional questions, e.g., if conversation stagnates

| Themes | Questions |
| --- | --- |
| 1 Introduction | Introduction of the interviewer, study objectives (enabling informed decisions), explanation of the process, clarification of any open questions  Brief explanation of the concept of digital mental health services (dMHSs) |
| 2 Starting question | How did you become aware of our study? (*Note: avoid clearly attributable information, otherwise blacken*). |
| 3 Knowledge about dMHSs | How high do you estimate your prior knowledge on the topic of digital mental health? And what do you base this on?  *** What dMHSs are you aware of (e.g., medical apps)? |
| 4 Student perspective and studies | Is the topic of (digital) mental health covered in your studies?  (To what extent) Has information about digital health or dMHSs been addressed and taught in your studies? (E.g., prescription of medical apps, application purposes, etc.).  ***If yes, how?  To what extent do you feel prepared for digitalization in your future profession (as a physician/psychologist) through your studies?  ***Do you know anything about existing (digital) mental health services at your university?  ***How do you think information about dMHSs should be disseminated on campus? (e.g., lecture, flyers, etc.).  ***What do you think about getting information about dMHSs from other students or lecturers?  Should information strategies on dMHSs be specifically targeted at medical/psychology students (or generally targeted at students)?  ***If yes, what information would you want specifically as a medical/psychology student? |
| 5 Experience with dMHSs | Have you used dMHSs *(such as apps for preventing stress or relaxation)* before?  ***Thinking about your experience with using dMHSs ...   - How did you become aware of the service? (how did you choose it?) - What did you like, what didn't you like about getting informed? What was particularly important to you? - Did your expectations of service XY come true? (Advertising or recommendation / information before use versus after experience with it). |
| 6 Information format | How would you look for dMHSs / how would you inform yourself about dMHSs? Which information format (virtual vs. non-virtual) do you prefer here?  ***Example to illustrate: imagine you are looking for a stress reduction app for a friend, where would you look for information on dMHSs? What media/information channels do you prefer to get relevant information quickly?  ***How much time are you willing to invest in the (first) search for information about the dMHS? (*Note: it is about first information, first impression*) |
| 7 Design preferences | What attracts your attention when you look for information, for instance on dMHSs? How should information be prepared (e.g., simple text form, texts with images, videos, podcast)?   - How should the texts/ videos/ images be presented in terms of design and layout? - What colors do you find appealing? - How should the information be prepared in terms of language? (e.g., funny, neutral tone, more specialized language) - How should the information be designed formally?   **What do you not like at all? (e.g., something like visually overwhelming, overloaded website, too much technical language) |
| 8 Content preferences | What information (in terms of content) is important to you in making a decision about using dMHSs, e.g. a medical app? What is most important? What information would prevent you from using dMHSs?   - What would you like to know in advance? What might influence your decision?   ***How important is information about … (e.g., data protection, costs, certain functions, etc.) to you when deciding whether to try out a dMHS or not?  ****Do you read user reviews and recommendations for products / dMHSs (e.g., purchase decision)? If yes, do you find other users’ experiences useful / helpful in your decision-making? (advantages, disadvantages) |
| 9 Information sources | Who is important to you as a source of information (e.g., physician, psychologist, friends, family, fellow students)? Who would have to recommend a dMHS to make you more likely to use it?  What sources of information do you find trustworthy? (*If no answer comes to mind, you could give examples such as health insurances, research journal, university, media, etc*.)  What exactly creates trust for you / makes information about e.g. apps credible? (if necessary, ask if „DiGA-Verzeichnis“ is known)?  *** What is your opinion of quality seals / certifications and why? Do you know any?  **** What opportunities and risks do you associate with digital mental health? Who is in the best position to judge this and should inform others about it? (e.g., experts, users, ...) |
| 10 Preparation for future role as HCP *(evaluated separately)* | How would you proceed as a HCP when you are informing yourself about new health services (especially in the area of mental health) for your patients?  What information would be important to you later in your career as a HCP when making decisions about whether to recommend or prescribe an app to a patient?  ****How would you like to get informed about medical innovations such as dMHSs as a HCP?  ****From your point of view as a future HCP, what creates trust / makes information about e.g. apps credible? |
| 11 Further wishes and suggestions | Do you have any suggestions or can you think of aspects we have not yet discussed?  ***In your point of view, what would the optimal information strategy on dMHSs for medical/psychology students look like?  Conclusion of the interview, thank you for participation, clarification of open questions, information on the further procedure |

Note: It is possible that the topic guide might change in the course of the data collection. This is common in qualitative research allowing to react to unexpected content.

**Notes**
